# Enhanced transcriptomic profiling of esophageal tissue through optimized PAXgene fixation protocols

**DOI:** 10.1016/j.gendis.2025.101842

**Published:** 2025-09-02

**Authors:** Fadi Charara, Louison Descampe, Ligia Craciun, Laurine Verset, Alexandre Spinette, Meriem Ennaji, Maarten Vander Kuylen, Calliope Maris, Pieter Demetter, Benjamin Beck

**Affiliations:** a Hôpitaux Universitaires de Bruxelles (HUB) – Institut Jules Bordet & Hôpital Erasme, Brussels 1070, Belgium; b Centre de Morphologie Pathologique (CMP), Brussels 1070, Belgium; c Laboratoire expérimental de gastroentérologie de l’Université Libre de Bruxelles, Brussels 1070, Belgium; d Institut de Recherche Interdisciplinaire en Biologie Humaine et Moléculaire (IRIBHM), Université Libre de Bruxelles (ULB), Brussels 1070, Belgium

Over the past decade, transcriptomics has emerged as a vital tool to study inter- and intra-sample heterogeneity. In acute myeloid leukemia, comparison of RNA sequencing with whole genome or exome sequencing has revealed that RNA sequencing enables identification of expressed gene fusions, single-nucleotide and short insertion/deletion variants, and whole-transcriptome expression information, thus offering the greatest diagnostic return.^1^ Furthermore, by elucidating distinct molecular subtypes, transcriptomics also enables the development of personalized therapeutic strategies.^2^^,^^3^

Transcriptomics necessitates deep sequencing to accurately capture all transcripts. Hence, the quality and integrity of samples form the bedrock of the entire procedure. However, formalin-based fixation, commonly utilized in clinical settings, is unfortunately recognized for its inability to preserve nucleotide integrity.^4^

Notably, investigating tumor–host interaction is crucial, but obtaining fresh tissue near surgical margins is limited by the need for prior pathological examination, restricting access to optimally preserved nucleic acids.

PAXgene fixation is designed to preserve nucleic acids while preserving tissue morphology of biological samples. However, there is a need for protocol customization according to the tissue in question, notably for those with high RNA degradation.

To assess the impact of fixation methods on RNA quality, we collected non-tumoral esophageal tissue from esophagectomy samples (*n* = 10), as this tissue type is known for its high susceptibility to RNA degradation. Each sample was divided into three portions and processed according to three different protocols: i) formalin fixation, ii) PAXgene fixation, or iii) flash freezing (Fig. 1A). The protocols for PAXgene-fixed paraffin-embedded (PFPE) and formalin-fixed paraffin-embedded (FFPE) samples are detailed in Table S1 and S2. We first assessed the impact of the type of tissue fixation on histomorphological criteria based on hematoxylin/eosin staining (Fig. 1B and C), and confirmed that PAXgene fixation was compatible with routine diagnosis of histopathologic conditions in esophageal tissue, as reported in the literature.^5^

**Figure 1:**
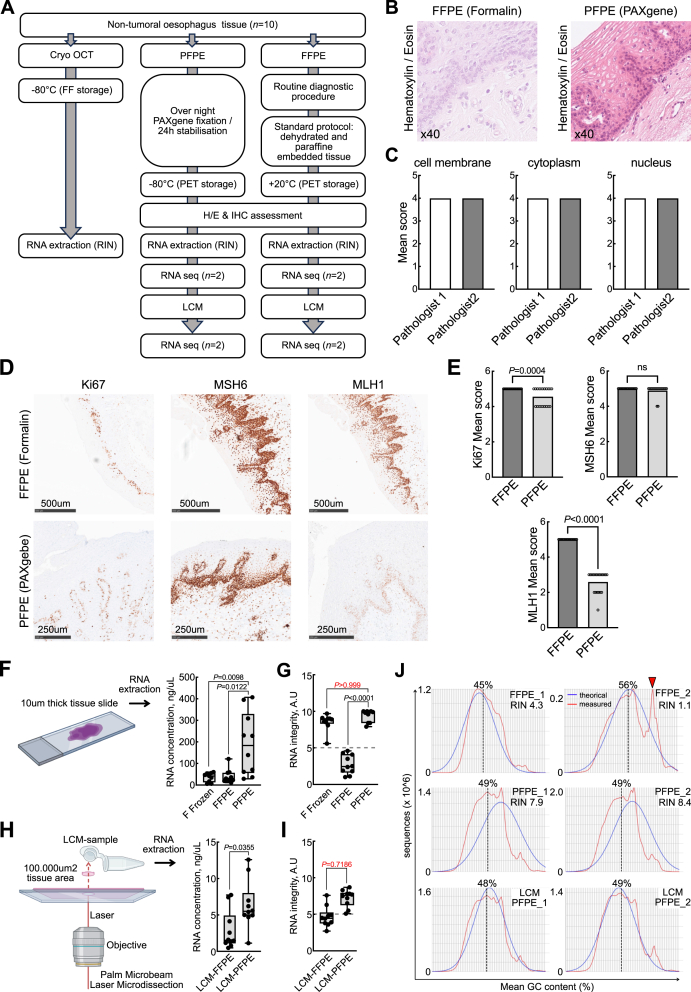
Preservation of esophagus tissue morphology and RNA integrity with PAXgene fixation. **(A)** Experimental design. **(B)** Hematoxylin/eosin staining of esophagus samples. Left: Fixed with formalin (FFPE); Right: Fixed with PAXgene (PFPE). Higher magnification images are displayed below. **(C)** The histogram showing the mean scores of cell morphology. The nuclear, cytoplasm, and cell membrane scores determined by two senior pathologists reached the highest scoring level (4/4) in both PFPE and FFPE samples. **(D)** Immunohistochemistry staining of Ki67, MSH6, and MLH1 in esophagus samples fixed with formalin (FFPE, upper picture) or PAXgene (PFPE, lower picture). **(E)** The histogram depicting immunohistochemical (IHC) scores of Ki67, MSH6, and MLH1 depending on the fixation method. **(F)** Comparison of RNA concentration in flash-frozen (*n* = 9), FFPE (*n* = 10), and PFPE (*n* = 10) samples. **(G)** Measurement of RNA integrity (RIN) in flash-frozen (*n* = 9), FFPE (*n* = 10), and PFPE (*n* = 10) samples. **(H)** RNA concentration in laser-capture microdissected (LCM) FFPE (*n* = 10) and LCM-PFPE (*n* = 10) samples. **(I)** Measurement of RNA integrity (RIN) in LCM-FFPE (*n* = 10) and LCM-PFPE (*n* = 10) samples. Statistics were calculated using non-parametric tests (Mann–Whitney for 2 conditions or Kruskal–Wallis for 3 conditions). **(J)** The per sequence GC content in RNA sequencing data in FFPE, PFPE, and LCM-PFPE conditions.

Then, we aimed at determining whether the PAXgene fixation method would affect common immunohistochemical analysis, used for diagnosis. For these immunohistochemical analyses, antibodies were selected based on their ubiquity. We focused on Ki67 and mismatch repair proteins (MutL homolog-1, MLH-1; MutS homolog 2, MSH2; MutS homolog 6, MSH6; postmeiotic segregation increased 2, PMS2). The intensity and specificity of immunohistochemical protein expression were scored by the same two pathologists according to a well-established scoring system (Fig. 1D and E). All markers tested showed positive staining in the FFPE samples, which were processed using standard pathology protocols. We then applied the same antibody panels to the matched PAXgene-fixed (PFPE) samples to evaluate whether this alternative fixation method would affect antigen preservation. We observed comparable staining for Ki67 and MSH6, reduced signal for MLH1, and absence of detectable staining for PMS2 and MSH2 in PFPE samples (Fig. S1). In conclusion, PAXgene fixation is compatible with the analysis of classical markers but may require modifications to the staining protocol of other markers such as PMS2 and MSH2.

Next, we assessed the impact of the fixation method on RNA integrity in all ten samples, in the three conditions (FFPE, PFPE, and flash-frozen), from a 10 μm thick slide. To this end, we extracted RNA with a dedicated kit matching the fixation protocol. First, we observed that FFPE samples had a poor profile when run on gel, with 18S and 28S ribosomal RNA peaks being barely detectable (Fig. S2A). PFPE samples on the opposite displayed clear peaks but were characterized by a high amount of small RNA, most likely miRNA (Fig. S2B). We therefore optimized the extraction protocol to mitigate the detrimental impact of miRNA spikes on RNA integrity score (RIN) calculation (Fig. S2C). The amendments in the tissue RNA/miRNA kit handbook are shown in Figure S2C.

The efficiency of the RNA extraction can be assessed by measuring the yield of the extraction as well as by calculating the RIN. Mean RNA yield from PFPE samples were greater than FFPE comparators (191 ng/μL (standard deviation/SD = 145) *vs*. 40.2 ng/μL (SD = 32.5); *P* = 0.0098; *n* = 10) but also than FF samples (35.8 ng/μL (SD = 21.3); *P* = 0.0122; *n* = 9) (Fig. 1F). More importantly, mean RIN extracted from PFPE samples was dramatically higher than FFPE comparators (9.39 (SD = 0.8185) *vs*. 2.72 (SD = 1.325); *P* < 0.0001) and similar to FF (8.5 (SD = 1.175); *P* > 0.99) (Fig. 1G).

To test whether further manipulation of the PFPE samples would affect RNA quality, we micro-dissected fixed areas of esophagus samples in FFPE and PFPE samples (Fig. 1H). Using our optimized extraction protocol, we obtained excellent RNA profiles with laser micro-dissected PFPE samples. Mean RNA yield from laser-capture microdissected PFPE (PFPE(L)) was lower than PFPE comparators (6.38 ng/uL (SD = 3.31) *vs*. 191 ng/uL (SD = 145)) but significantly higher than laser-capture microdissected FFPE (FFPE(L)) samples (2.87 ng/uL (SD = 0.85); *P* = 0.0355) (Fig. 1H). Mean RIN measured in PFPE(L) was not significantly higher than FFPE(L) comparators (7.21 (SD = 1.169) *vs*. 4.64 (SD = 1.406); *P* = 0.7186) (Fig. 1I). Nonetheless, 8 out of 10 FFPE(L) samples had a RIN value inferior to 5 (threshold under which the genomic facility recommends not to sequence RNA samples), while all the PFPE(L) samples had a RIN value superior to 5, pointing to a significant difference in the proportion of samples that are eligible to RNA sequencing in both conditions (Fisher's exact test; *P* = 0.0007). For better readability, the RNA extraction results are reported in Table S3.

To determine whether the fixation method would influence RNA sequencing data, we randomly selected the samples resulting in one of the highest and lowest RIN in FFPE (RIN = 4.3 and 1.1), paired PFPE samples (RIN = 7.9 and 8.4), and random PFPE(L) samples (RIN = 7.3 and 6.8) conditions. We sequenced these six samples and observed a high per-sequence GC content (mean GC content = 56%) in the sample with the lowest RIN (FFPE_2), whereas the mean GC content was about 50% in the other samples. In addition, a sharp peak on the normal distribution of the FFPE_2 sample suggests over-represented sequences (Fig. 1J). Interestingly, RNA expression values were similar for PFPE and PFPE(L) samples, whereas the FFPE sample with the lowest RIN differed significantly from the others (Fig. S3). Consequently, RNA sequencing data from PFPE and PFPE(L) samples showed high similarity (*r* > 0.94), in contrast to the FFPE samples, which were less consistent (*r* = 0.864). These data show that PAXgene fixation is compatible with RNA sequencing and that laser microdissection does not affect RNA integrity to a point that jeopardizes RNA profiling.

Given that FFPE tissue remains the gold standard in clinical pathology, integrating emerging fixation methods, such as PFPE, requires a thoughtful, phased approach. One practical strategy to bridge these methods involves dual processing, in which parallel sections or biopsies from the same tissue sample are fixed using both FFPE and PFPE protocols. This allows FFPE samples to be used for routine histopathological assessment, while reserving PFPE material for high-quality molecular analyses, particularly in contexts where nucleic acid preservation is critical, such as transcriptomic profiling or mutation screening.

Broader clinical adoption will depend on harmonizing protocols and diagnostic criteria across fixation methods, ensuring diagnostic reproducibility, and addressing regulatory requirements. In conclusion, our findings confirm that PAXgene fixation is compatible with both morphological and molecular analyses in esophageal samples, offering a valuable complement to FFPE in clinical workflows.

## CRediT authorship contribution statement

**Fadi Charara:** Conceptualization, Writing – original draft, Methodology, Data curation, Project administration, Investigation. **Louison Descampe:** Formal analysis, Investigation, Data curation. **Ligia Craciun:** Investigation, Methodology, Conceptualization. **Laurine Verset:** Investigation, Formal analysis. **Alexandre Spinette:** Investigation. **Meriem Ennaji:** Investigation. **Maarten Vander Kuylen:** Investigation. **Calliope Maris:** Visualization, Investigation. **Pieter Demetter:** Supervision, Investigation, Validation, Writing – review & editing. **Benjamin Beck:** Supervision, Formal analysis, Validation, Funding acquisition, Writing – review & editing, Methodology.

## Ethics declaration

This study was approved by the local ethics committee of the Institut Jules Bordet (HUB)-ECPSO/LEC with the internal reference CE3344.

## Data availability

The data that support this study are available from the corresponding author upon request. Bulk RNA sequencing data will be deposited in the NCBI Gene Expression Omnibus if the manuscript is accepted for publication.

## Funding

B.B. is funded by 10.13039/501100002661Fonds De La Recherche Scientifique - FNRS (No. 40021386 – PDR, 40028944 – CDR) and The Belgian Fondation contre le cancer (No. F/2022/1932). L.D. is supported by a Télévie PhD fellowship (No. 40007654).

## Conflict of interests

The authors declare the following financial interests/personal relationships which may be considered as potential competing interests: Beck reports financial support was provided by The Belgian National Fund for Scientific Research (FNRS). Beck reports financial support was provided by The Belgian Fondation contre le cancer. Descampe reports financial support was provided by Télévie PhD fellowship. If there are other authors, they declare that they have no known competing financial interests or personal relationships that could have appeared to influence the work reported in this paper.

## References

[1] Docking T.R., Parker J.D.K., Jädersten M. (2021). A clinical transcriptome approach to patient stratification and therapy selection in acute myeloid leukemia. Nat Commun.

[2] Nolan E., Lindeman G.J., Visvader J.E. (2023). Deciphering breast cancer: from biology to the clinic. Cell.

[3] Culhane A.C., Howlin J. (2007). Molecular profiling of breast cancer: transcriptomic studies and beyond. Cell Mol Life Sci.

[4] Srinivasan M., Sedmak D., Jewell S. (2002). Effect of fixatives and tissue processing on the content and integrity of nucleic acids. Am J Pathol.

[5] Barroux M., Horstmann J., Fricke L. (2023). Histological evaluation of PAXgene tissue fixation in Barrett's esophagus and esophageal adenocarcinoma diagnostics. Virchows Arch.

