# Supplementary material for: Enhanced transcriptomic profiling of esophageal tissue through optimized PAXgene fixation protocols
Source: Genes Dis. 2025 Sep 2;13(3):101842. doi: 10.1016/j.gendis.2025.101842 (PMC12855549; doi:10.1016/j.gendis.2025.101842)
Supplement: Multimedia component 1 [file mmc1.docx]

**SUPPLEMENTARY DATA:**

**MATERIAL & METHODS**

Samples

Tissue samples were collected from esophagectomies performed for cancer treatment (*n*=10). In line with guidelines recommending an eight cm margin from the tumor during oesophageal surgery, we obtained 3cm^2^ samples of healthy tissue, up to the submucosa, from a position five cm proximal to the lesion. This sampling strategy was designed to preserve the circular margin and avoid interference with tumor and longitudinal margin analysis. Each tissue sample was immediately divided into three portions and processed according to three different protocols. For the fresh frozen samples, the tissue was immediately encased in a Cryomold-intermediate (15 mm x 15 mm x 5 cm) Tissue-Tek Sakura, covered by OCT™, and flash-frozen at -80°C. The protocols for PAXgene-fixed paraffin-embedded (PFPE) and formalin-fixed paraffin-embedded (FFPE) samples are detailed in Table 1&2.

Morphology and immunohistochemistry

For clinical relevance, the reliability of morphology and immunohistochemistry must match that of standard FFPE tissue. We thus prepared 4 µm thick slides from both the FFPE and PFPE samples, subjecting them to hematoxylin eosin staining. The slides were independently reviewed by two senior pathologists (PD&LV), with morphological evaluation carried out based on histomorphology scoring criteria^1^.

For the immunohistochemical analysis, antibodies were selected based on their ubiquity in tissue expression. We focused on mismatch repair proteins (MLH-1, MSH2, MSH6, PMS2) and Ki67. The intensity and specificity of IHC protein expression were scored by the same two pathologists according to a scoring system published by Southwood et al^2^. Deparaffinization and antigen activation of the 4-μm-thick serial sections of FFPE and PFPE tissue were achieved by heat treatment at high pH.(8,4) The IHC process was conducted using the OptiView DAB IHC Detection kit (Ventana), an automated staining instrument (BenchMark *ULTRA IHC/ISH System, Roche*), and primary monoclonal antibodies against anti-MLH1 (clone ES05, “ready to use”, Dako/Agilent), anti-MSH2 (clone FE11, “ready to use”, Dako/Agilent), anti-MSH6 (clone EP49, “ready to use”, Dako/Agilent), anti-PMS2 (clone EPR3947, “ready to use”, Cell Marque), and anti-KI67 (clone 30-9, “ready to use”, Roche).

Image data acquisition and digital image analysis

All slides were scanned using a Nanozoomer S360 Hamamatsu^®^. QuPath software version 0.3.2 (Bankhead, P. et al., 2017) was employed for digital pathology imaging analysis. First, Ki67-positive cells were identified using the Cell detection command. Subsequently, by using a single measurement classifier with a nuclear eosin optical density mean threshold of 0.15 for FFPE slides and 0.08 for PFPE slides, cells were classified as either low or high Ki67 positive.

RNA extraction

RNA extraction was performed following manufacturer’s protocol, namely “Purification of Total RNA, Including miRNA, from Sections of PFPE Tissue” (PAXgene® Tissue RNA/miRNA Kit Handbook). As described in the main text, two different methods were used to precipitate RNA: Isopropanol 100% (as recommended in the original protocol) or Ethanol 70% to decrease the amount of miRNA.

Analyses of nucleotide preservation

We assessed nucleotide preservation in all ten samples, each preserved using the three different protocols, and processed from a 10 µm thick slide. Each RNA extraction was performed with a dedicated kit, specifically matched to the fixation protocol used: the PAXgene® Tissue miRNA Kit (*Qiagen*) for PFPE, the miRNeasy FFPE Kit (*Qiagen*) for FFPE, and the miRNeasy Micro Kit (*Qiagen*) for fresh frozen samples.

Laser Microdissection

Laser microdissection was performed using a Palm Microbeam Laser Microdissection, Zeiss^®^, Germany. From same each ten PFPE and FFPE preparations, we cut a 10 µm thickness slide in RNase free conditions (MembraneSlide NF 1.0 PEN, ZEISS, 415190-9081-001) impregnated with RNAse-free water (Ambion Nuclease water, AM9937) and dry overnight at room temperature. Slides were deparaffinized and stained with hematoxylin alone since eosine may further degrade RNA.

First, slides were immersed in two changes of xylene (Technical, 28973.294) during 2 minutes.

Following deparaffination, slides were subsequently immersed in decreasing concentrations of ethanol bath (100%, 96%, 70%, Ensyre, 1.009835000) and stored at -80°C.

Dissection was performed on the epithelial sample’s side on a total of 4 million µm^2^. This area was obtained by the addition of multiples small uniform 100.000 µm^2^ squares. Robot settings used was strictly the same during the experiment except the cycle number applications (table 3).

RNA sequencing

Indexed cDNA libraries were obtained using the Ovation Solo RNA-Seq Library Preparation Kit (Tecan, Männedorf, Switzerland) following manufacturer’s recommendations. The multiplexed libraries were loaded on a NovaSeq 6000 (Illumina, San Diego, CA, USA) using a S2 flow cell and sequences were produced using a 200 Cycle Kit. Paired-end reads were mapped against the human reference genome GRCh38 using STAR software (version 2.5.3a) to generate read alignments for each sample. Annotations Homo_sapiens.GRCh38.90.gtf were obtained from ftp.Ensembl.org. The metrics and the quality of our sequencing data were assessed using FastQC (http://www.bioinformatics.babraham.ac.uk/projects/download.html#fastqc). After transcripts assembling, gene level counts were obtained using HTSeq-0.9.1 ^3^.

We then analyzed the RNA sequencing data using *iDep*^4^.

Statistics

Results are expressed as the means + standard deviation (s.d) when indicated. Box plots represent median, lower and upper quartiles and whiskers represent minimal to maximal values. All values are represented on the graphs. Statistical analyses were performed using non parametric tests (*Mann-Whithey* when comparing 2 samples or *Kruskal-Wallis* followed by *Dunn*’s multiple comparison when comparing 3 samples or more).

A value of *P*<0.05 was considered to be statistically significant. All calculations were performed using GraphPad Prism Software version 10 (*GraphPad*).

1. Craft, W. F., Conway, J. A. & Dark, M. J. Comparison of histomorphology and DNA preservation produced by fixatives in the veterinary diagnostic laboratory setting. *Peerj* **2**, e377 (2014).

2. Southwood, M. *et al.* Systematic evaluation of PAXgene® tissue fixation for the histopathological and molecular study of lung cancer. *J Pathology Clin Res* **6**, 40–54 (2020).

3. Anders, S., Pyl, P. T. & Huber, W. HTSeq—a Python framework to work with high-throughput sequencing data. *Bioinformatics* **31**, 166–169 (2015).

4. Ge, S. X., Son, E. W. & Yao, R. iDEP: an integrated web application for differential expression and pathway analysis of RNA-Seq data. *BMC Bioinform.* **19**, 534 (2018).
