# Supplementary material for: Enhanced transcriptomic profiling of esophageal tissue through optimized PAXgene fixation protocols
Source: Genes Dis. 2025 Sep 2;13(3):101842. doi: 10.1016/j.gendis.2025.101842 (PMC12855549; doi:10.1016/j.gendis.2025.101842)
Supplement: Multimedia component 2 [file mmc2.docx]

**Figure S1** Compatibility of PAXgene fixation with several classical immunostaining. **(A)** Immunohistochemistry staining of Ki67 (marker of proliferation Kiel 67) in esophagus samples fixed with formalin (FFPE, left) or PAXgene (PFPE, right). **(B)** Comparison of Ki67 staining in samples fixed with the two different methods. **(C)** Evaluation of Ki67 staining in samples fixed with the two different methods using digital pathology imaging analysis. **(D)** Immunohistochemistry staining of PMS2 in esophagus samples fixed with formalin (FFPE, left) or PAXgene (PFPE, right). The histogram on the right depicts the immunohistochemical (IHC) score of PMS2 depending on the fixation method. **(E)** Immunohistochemistry staining of MSH2 in esophagus samples fixed with formalin (FFPE, left) or PAXgene (PFPE, right). The histogram on the right depicts the IHC score of MSH2 depending on the fixation method. **(F)** Immunohistochemistry staining of MSH6 in esophagus samples fixed with formalin (FFPE, left) or PAXgene (PFPE, right). **(G)** Immunohistochemistry staining of MLH1 in esophagus samples fixed with formalin (FFPE, left) or PAXgene (PFPE, right). The subset of pictures in (A), (F), and (G) is shown in Figure 1D.

**Figure S2** RNA quality of PAXgene-fixed esophagus samples. RNA profile analysis was conducted using the bioanalyzer automated electrophoresis. **(A)** RNA-poor profile in FFPE tissue fixation. **(B)** RNA profile with clear 18S and 28S ribosomal RNA peaks, most likely miRNA in PFPE tissue fixation. **(C)** The RNA profile resulting from the amendment of "tissue RNA/miRNA kit handbook" as follow: the 650 μL addition of xylene and ethanol 100% was suppressed at step 3 to step 6, isopropanol was replaced by ethanol 70% at step 12 and step 25 was suppressed except if RNA structure was complex, and 65 °C incubation lasted 30 s.

**Figure S3** RNA quality of PAXgene-fixed esophagus samples is compatible with next-generation sequencing. **(A)** Experimental design. Bulk RNA sequencing was conducted on FFPE, PFPE, and laser-capture microdissected (LCM) PFPE samples, selected based on their highest and lowest RNA integrity number (RIN). The shaded area represents the threshold below which the core facility does not recommend sequencing RNA samples (RIN < 5). These data are also shown in Figure 1I, K. **(B)** The distribution of transformed RNA sequencing data in FFPE, PFPE, and LCM-PFPE conditions. **(C)** Density plot of the distribution of RNA sequencing transformed data in FFPE, PFPE, and LCM- PFPE conditions. **(D)** Scatter plot of transformed expression. This scatter plot compares the transformed expression in two samples from FFPE, PFPE, and LCM-PFPE conditions. The *r* correlation coefficient is indicated for each condition. This data shows the apparent loss of data for transcripts with low expression values in RNA extracted from FFPE samples.
