# Supplementary material for: Enhanced transcriptomic profiling of esophageal tissue through optimized PAXgene fixation protocols
Source: Genes Dis. 2025 Sep 2;13(3):101842. doi: 10.1016/j.gendis.2025.101842 (PMC12855549; doi:10.1016/j.gendis.2025.101842)
Supplement: Multimedia component 3 [file mmc3.pdf]

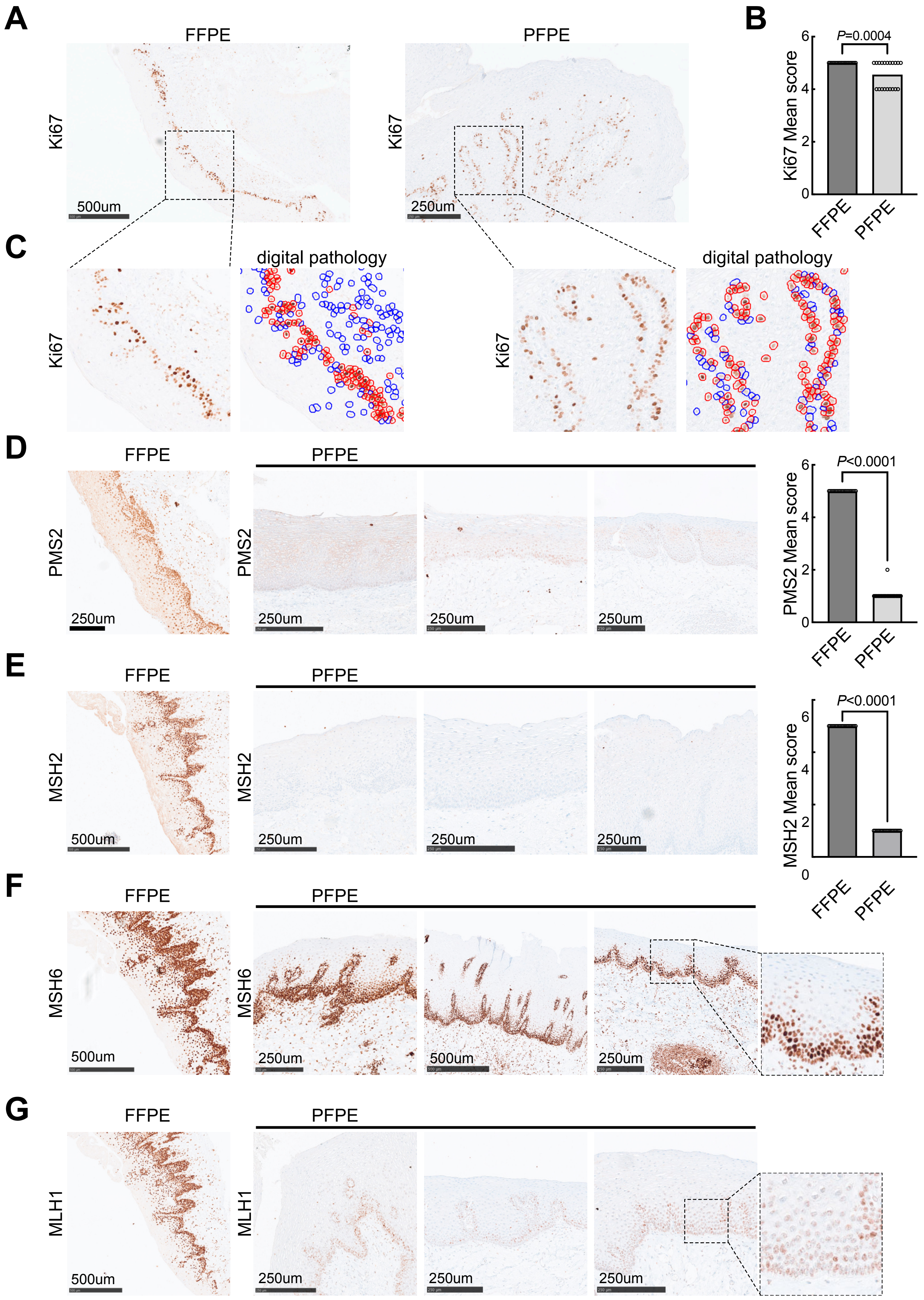

Supplementary Figure1. Compatibility of PAXgene fixation with several classical immunostaining

(**A**) Immunohistochemistry staining of Ki67 (marker of proliferation Kiel 67) in oesophagus samples fixed with Formalin (FFPE, left) or PAXgene (PFPE, right). (**B**) Comparison of Ki67 staining in samples fixed with the two different methods. (**C**) Evaluation of Ki67 staining in samples fixed with the two different methods using digital pathology imaging analysis. (**D**). Same as in (A) with PMS2. Histogram on the right depicts PMS2 IHC score depending on the fixation method. (**E**) Same as in (A) with MSH2. Histogram on the right depicts MSH2 IHC score depending on the fixation method. (**F**) Same as in (A) with MSH6. (**G**) Same as in (A) with MLH1. Subset of pictures in A, F and G are shown in the Main Figure1D.
