# Supplementary material for: Enhanced transcriptomic profiling of esophageal tissue through optimized PAXgene fixation protocols
Source: Genes Dis. 2025 Sep 2;13(3):101842. doi: 10.1016/j.gendis.2025.101842 (PMC12855549; doi:10.1016/j.gendis.2025.101842)
Supplement: Multimedia component 4 [file mmc4.pdf]

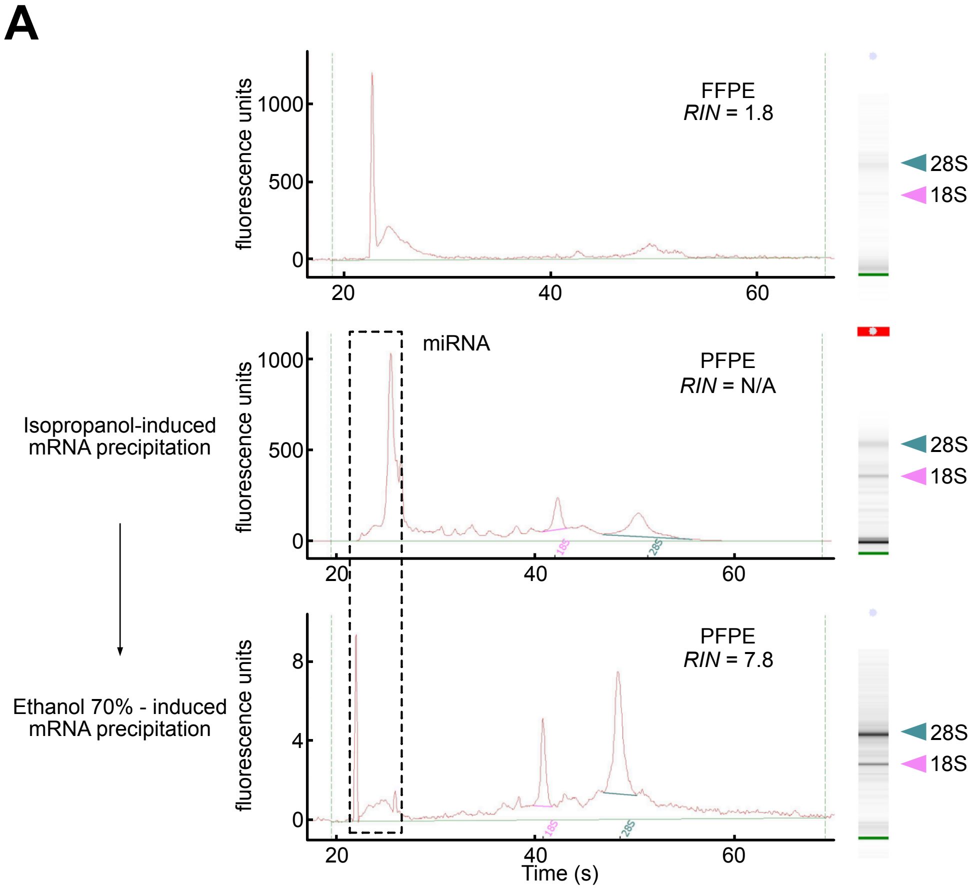

Supplementary Figure2. RNA Quality of PAXgene-Fixed Esophagus Samples

(**A**) RNA profile analysis conducted using bioanalyzer automated electrophoresis (raw image is displayed on the right)**.**
