# Supplementary material for: Enhanced transcriptomic profiling of esophageal tissue through optimized PAXgene fixation protocols
Source: Genes Dis. 2025 Sep 2;13(3):101842. doi: 10.1016/j.gendis.2025.101842 (PMC12855549; doi:10.1016/j.gendis.2025.101842)
Supplement: Multimedia component 5 [file mmc5.pdf]

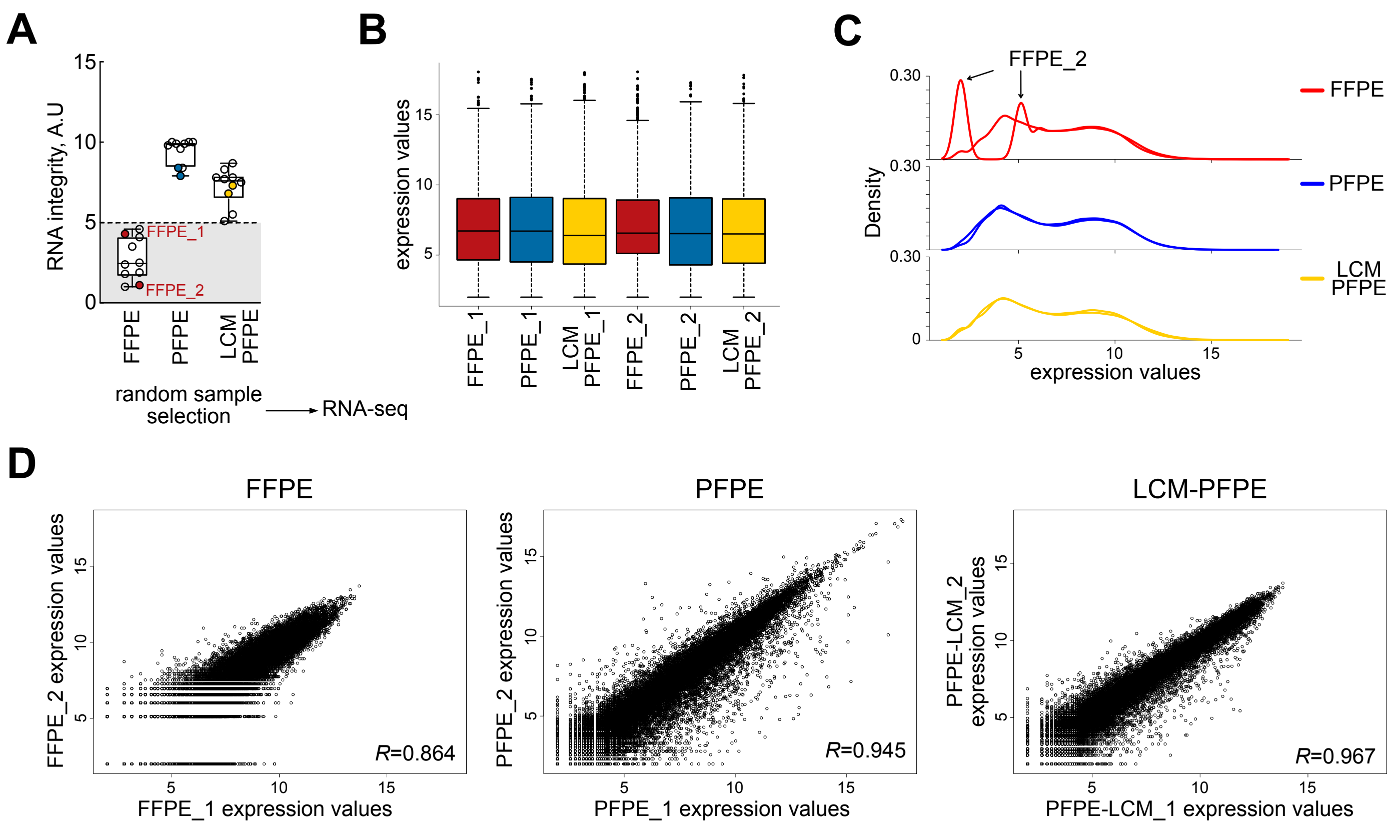

Supplementary Figure3: RNA Quality of PAXgene-Fixed Esophagus Samples Compatible with Next-Generation Sequencing

(**A)** Experimental Design. Bulk RNA sequencing was conducted on FFPE, PFPE, and laser-capture microdissected (LCM) PFPE samples, selected based on their highest and lowest RNA Integrity Number (RIN) values. The shaded area represents the threshold below which the core facility does not recommend sequencing RNA samples (RIN < 5). These data are also shown in Fig.1I, K. (**B**) This panel illustrates the distribution of transformed RNA-seq data in FFPE, PFPE, and LCM-PFPE conditions. (**C**) Density Plot visualizing the distribution of RNA-seq transformed data in FFPE, PFPE, and LCM-PFPE conditions. (**D**). Scatter Plot of Transformed Expression. This scatter plot compares the transformed expression in two samples from FFPE, PFPE, and LCM-PFPE conditions. The R correlation coefficient is indicated for each condition. This data shows the apparent loss of data for transcripts with low expression values in RNA extreacted from FFPE samples.
