# Supplementary material for: Enhanced transcriptomic profiling of esophageal tissue through optimized PAXgene fixation protocols
Source: Genes Dis. 2025 Sep 2;13(3):101842. doi: 10.1016/j.gendis.2025.101842 (PMC12855549; doi:10.1016/j.gendis.2025.101842)
Supplement: Multimedia component 6 [file mmc6.docx]

**Table S1. *FFPE fixation Protocol***

| Step | Media | Time | Temperature |
| --- | --- | --- | --- |
| 1 | Formol | 150 min | 37°C |
| 2 | Alcool 70° | 60 min | 37°C |
| 3 | 97% ethanol | 30 min | 37°C |
| 4 | 97% ethanol | 60 min | 37°C |
| 5 | isopropanol | 60 min | 37°C |
| 6 | isopropanol | 60 min | 37°C |
| 7 | isopropanol | 60 min | 37°C |
| 8 | Xylene | 60 min | 37°C |
| 9 | Xylene | 60 min | 37°C |
| 10 | Xylene | 60 min | 37°C |
| 11 | Xylene | 60 min | 37°C |
| 12 | Paraffin | 45min | 62°C |
| 13 | Paraffin | 45min | 62°C |
| 14 | Paraffin | 45min | 62°C |

**Table S2. *PAXgene fixation Protocol***

| Step | Media | Time | Temperature |
| --- | --- | --- | --- |
| 1 | PAXgene Tissue fixation | Min 14h - Max 19h | 18-22°C |
| 2 | PAXgene Tissue STABILIZER | Min 20h - Max 24h | 18-22°C |
| 3 | 99% ethanol | 30 min | 18-22°C |
| 4 | 99% ethanol | 30min | 18-22°C |
| 5 | 99% ethanol | 30min | 18-22°C |
| 6 | 99% ethanol | 30 min | 18-22°C |
| 7 | 99% ethanol | 30 min | 18-22°C |
| 8 | Isopropanol | 30 min | 18-22°C |
| 9 | Isopropanol | 30 min | 18-22°C |
| 10 | Xylene | 30 min | 18-22°C |
| 11 | Xylene | 60 min | 18-22°C |
| 12 | Paraffin | 135-150min | 62°C |

**Table S3. *RNA extraction results***

|  | RNA integrity (RIN) | | | | | RNA concentration (ng/uL) | | | | |
| --- | --- | --- | --- | --- | --- | --- | --- | --- | --- | --- |
| Sample | FFPE | **P**FPE | FF | FFPE **L** | **P**FPE **L** | FFPE | **P**FPE | FF | FFPE **L** | **P**FPE **L** |
| 1 | 1,8 | 10 | 8.6 | 4,5 | 7,5 | 23 | 61 | 60 | 1,47 | 6,2 |
| 2 | 4,1 | 9,9 | 8,9 | 4,8 | 7,8 | 36 | 230 | 47 | 1,59 | 1,13 |
| 3 | 4,3 | 7,9 | 8,5 | 7,6 | 7,3 | 57 | 71 | 17 | 2,57 | 11,22 |
| 4 | 2,4 | 9,9 | 9,7 | 3,6 | 8,3 | 11 | 29 | 5,45 | 4,13 | 12,6 |
| 5 | 3,5 | 10 | 8,1 | 6,3 | 7,7 | 121 | 407 | 42 | 0,48 | 5 |
| 6 | 1,1 | 8,4 | 9 | 4,9 | 7,8 | 30 | 395 | 56 | 4,13 | 4,7 |
| 7 | 2,5 | 9,8 | 9,1 | 2,7 | 6,8 | 59 | 36 | 6,24 | 0,786 | 6,98 |
| 8 | 4,6 | 8,4 | 9 | 3,8 | 5,1 | 21 | 309 | 54 | 7,74 | 5,42 |
| 9 | 1.9 | 10 | 5,6 | 4,4 | 5,5 | 25 | 139 | 34,57 | 1,38 | 5,6 |
| 10 | 1 | 9,6 | m | 3,9 | 8,7 | 19 | 228 | 1,17 | 7,43 | 4,95 |
